# Supplementary material for: Injection into and extraction from single fungal cells
Source: Commun Biol. 2022 Mar 1;5:180. doi: 10.1038/s42003-022-03127-z (PMC8888671; doi:10.1038/s42003-022-03127-z)
Supplement: Supplementary file 2 — Supplementary Information [file 42003_2022_3127_MOESM2_ESM.pdf]

Supplementary Information

Injection into and extraction from single fungal cells

Orane Guillaume-Gentil,[1*] Christoph G. Gäbelein,[1] Stefanie Schmieder,[1,#] Vincent Martinez,[2] Tomaso Zambelli,[2] Markus Künzler,[1] Julia A. Vorholt[1*]

[1]Institute of Microbiology, ETH Zurich, 8093 Zurich, Switzerland; [2]Institute for Biomedical Engineering, ETH Zurich, 8092 Zurich, Switzerland

[#]current address: Division of Gastroenterology, Boston Children's Hospital, Boston, MA 02115, USA

*Correspondence: gorane@ethz.ch, jvorholt@ethz.ch

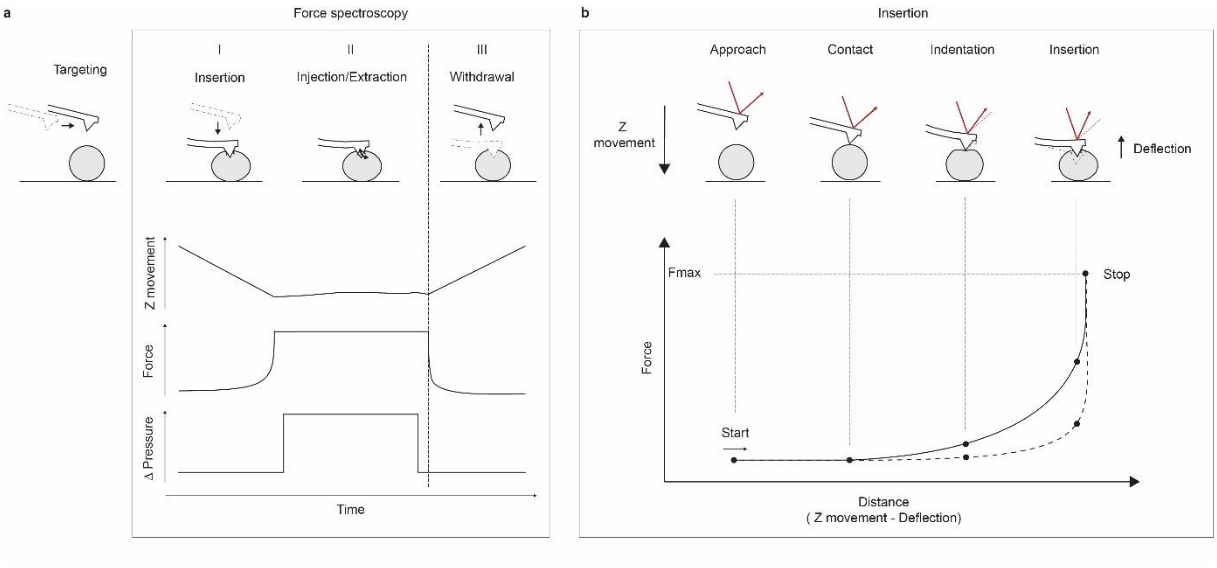

Supplementary Figure 1. Automated force spectroscopy approach for injection and extraction of fungi. a) The routine consists in 3 successive phases. In the first phase, the probe is first driven down at constant speed (1000 nm s^{-1}) while the force sensed at the tip is recorded; the probe movement is stopped when a pre-defined force set-point is reached (maximal force, up to 2000 nN); no pressure is applied through the microchannel; phase I lasts less than 10 s. In the second phase, the force is kept constant at the pre-set maximal force, and vertical movement of the probe allows for compensating eventual drifts; shortly after stabilization of the tip inside the cell, a positive pressure difference is applied through the microchannel for injection; injection is stopped by switching the pressure back to zero; this second phase lasts several minutes. In the third phase, the probe is withdrawn out of the cell at constant speed; the force is recorded, and no pressure is applied; the phase lasts less than 10 s. b) Probe insertion. Force-Distance profiles are obtained from phase I, in which the measured force is plotted against the Z-movement of the probe (down) to which the probe deflection (up) is subtracted. When the probe is driven towards the selected cell, the force sensed by the tip is zero until it contacts the cell; the force then start to gradually increase as the tip is pushed further down, indenting the cell; pushing further down, the tip crosses through the cell wall and membrane. The cell wall rupture events are not detected, due to the high stiffness of the probe. The stiffer is the cell, the higher are the forces exerted on the tip upon indentation and insertion; the solid line thus reflects a stiffer cell compared to the dashed line.

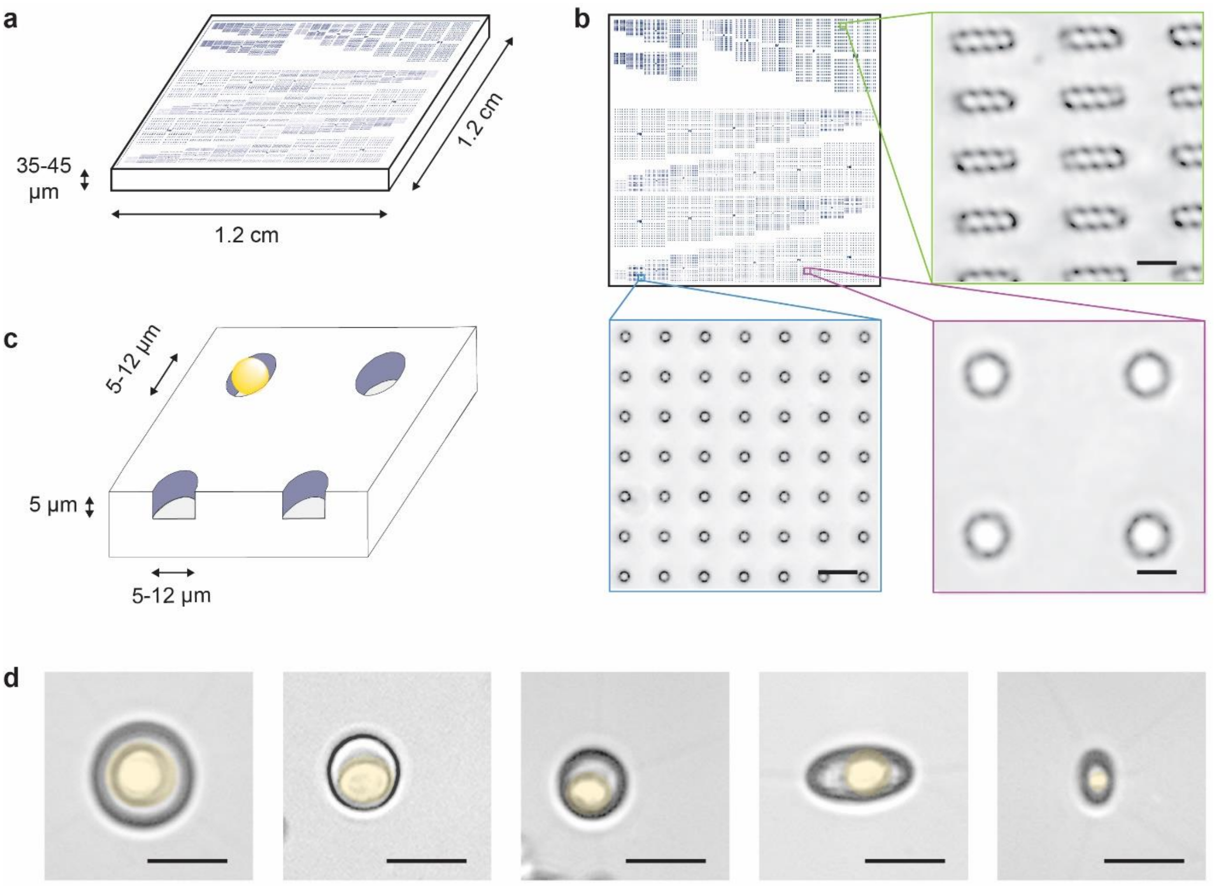

Supplementary Figure 2. Micropatterned substrates used to immobilize *S. cerevisiae* cells for injection experiments. a) The microstructure arrays are 1.2 x 1.2 cm^2, with a thickness of 40 ± 5 µm. b) The arrays features round and oval microwells, with dimensions varying from 2 up to 30 µm. Microwell depths ranging from 2 up to 15 µm were tested. Scale bars are 20 µm. c) Effective yeast constriction for injection by FluidFM was obtained with round and oval microwells with a depth of 5 µm, and lateral dimensions ranging between 5 and 12 µm. d) Representative brightfield microscopy images of individual *S. cerevisiae* (yellow overlay) effectively constrained in microwells. Scale bars are 10 µm.

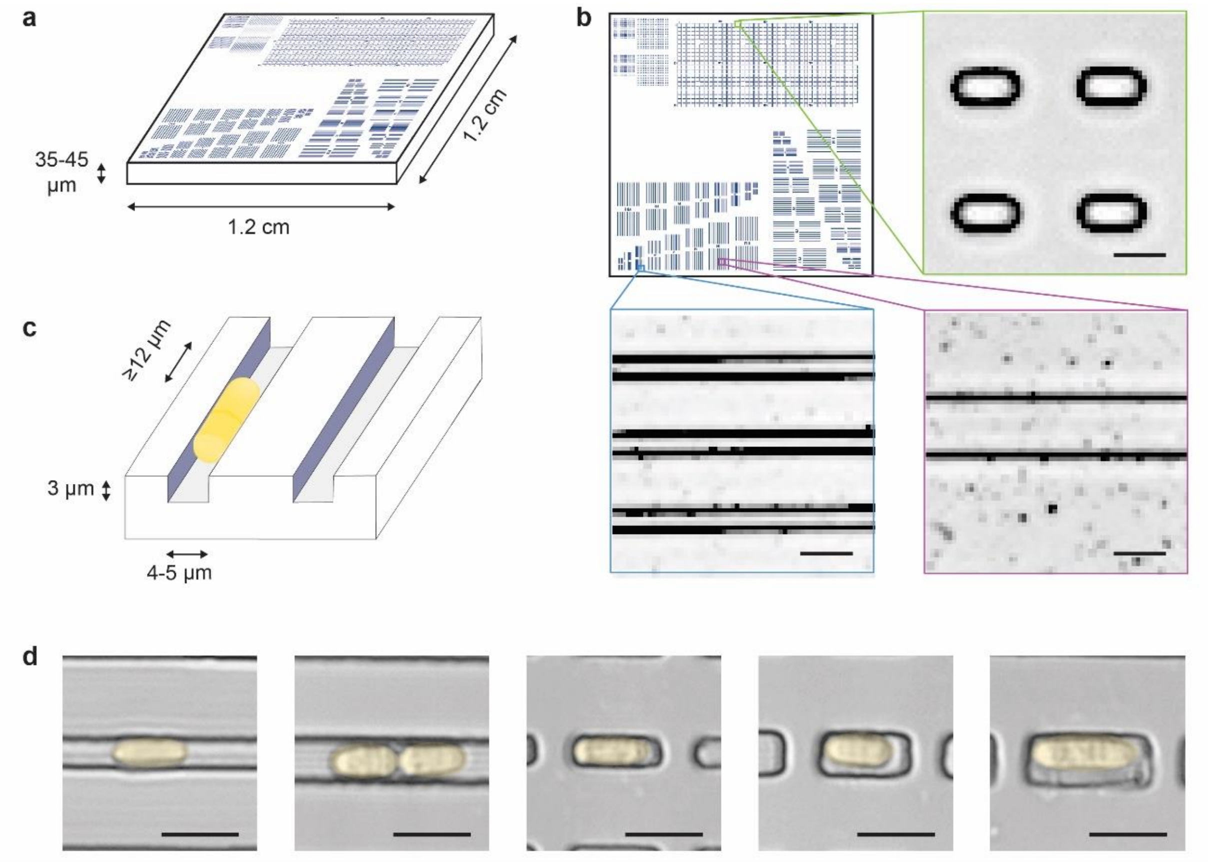

Supplementary Figure 3. Micropatterned substrates used to immobilize *S. pombe* cells for injection experiments. a) The microstructure arrays are 1.2 x 1.2 cm^2, with a thickness of 40 ± 5 µm. b) The arrays features trenches and rectangle and oval microwells, with width ranging from 2 to 30 µm. Microwell depths ranging from 1 up to 10 µm were tested. The scale bars on the brightfield images are 20 µm. c) Effective yeast constriction for injection by FluidFM was obtained with trenches and microwells with a depth of 3 µm, a width of 4-5 µm, and length of 12 µm or longer. d) Representative brightfield microscopy images of individual *S. pombe* (yellow overlay) effectively constrained in trenches and microwells. Scale bars are 10 µm.

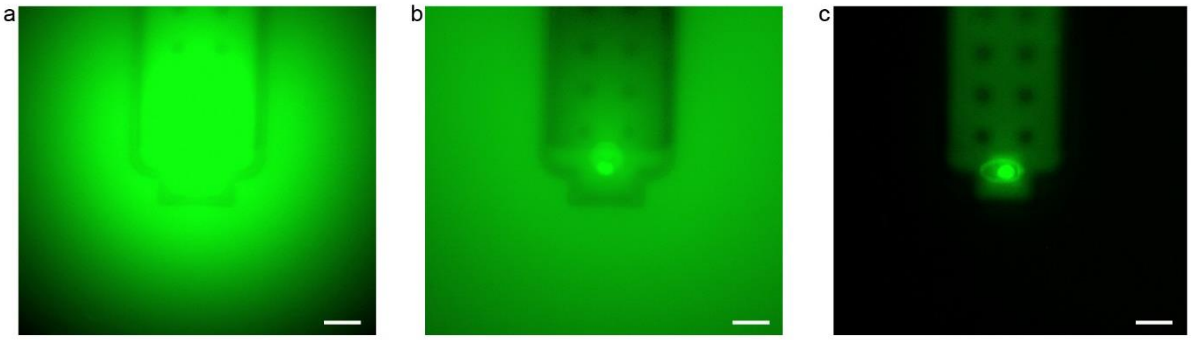

Supplementary Figure 4. *In situ* fluorescence imaging upon injection of *S. cerevisiae*. Extracellular leakage of the fluorophore is readily observed in cases where the probe aperture is not inserted (a), or only partially inserted (b) in the cell interior. Upon complete insertion of the probe aperture inside the cell, cell staining but no leakage are observed (c). All scale bars are 10 µm.

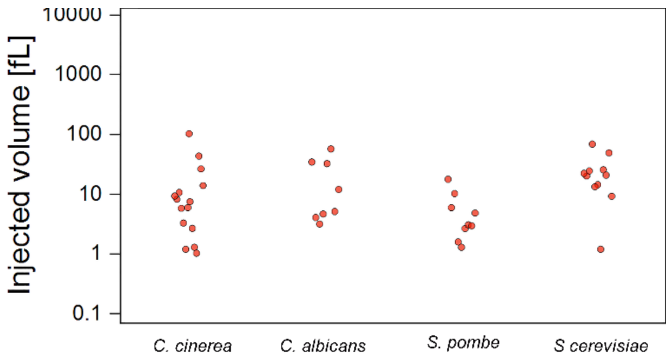

Supplementary Figure 5. Injected volumes as measured from intracellular fluorescence of Lucifer yellow in *C. cinerea* (N=15), *C. albicans* (N=8), *S. pombe* (N=9) and *S. cerevisiae* (N=11).
