# Supplementary material for: Injection into and extraction from single fungal cells
Source: Commun Biol. 2022 Mar 1;5:180. doi: 10.1038/s42003-022-03127-z (PMC8888671; doi:10.1038/s42003-022-03127-z)
Supplement: Supplementary file 3 — Description of Additional Supplementary Files [file 42003_2022_3127_MOESM3_ESM.pdf]

Description of Additional Supplementary Files

File name: Supplementary Data
Description: Source data for the graphs and charts presented in this manuscript.
